# Rare Variant of Hereditary Amyloid Transthyretin Cardiomyopathy Secondary to Ser97Tyr Mutation

**DOI:** 10.7759/cureus.65891

**Published:** 2024-07-31

**Authors:** Siva Naga S Yarrarapu, Tarun Dalia, Ilham Boda, Amandeep Goyal, Andrija Vidic, Zubair Shah

**Affiliations:** 1 Internal Medicine, Monmouth Medical Center, Long Branch, USA; 2 Cardiology/Advanced Heart Failure, University of Kansas, Kansas City, USA; 3 Cardiology, University of Kansas Medical Center, Kansas City, USA; 4 Cardiology/Advanced Heart Failure, University of Kansas Medical Center, Kansas City, USA

**Keywords:** transthyretin cardiac amyloidosis, cardiac amyloidosis, hereditary transthyretin amyloidosis, transthyretin cardiomyopathy, transthyretin amyloidosis

## Abstract

Hereditary transthyretin amyloidosis (hATTR) is an autosomal dominant, adult-onset disease that stems from point mutations in the TTR gene encoding the protein transthyretin. The disease is progressive and life-threatening and is associated with amyloid deposits in multiple organs including the heart, kidney, skin, eyes, nervous system, and gastrointestinal tract. Genotypic and phenotypic heterogeneity is a characteristic hallmark of hereditary transthyretin amyloidosis. Herein, we present a rare variant of hATTR cardiomyopathy secondary to Ser97Tyr mutation, having been documented only in a handful of families previously. This case serves as a valuable opportunity to elucidate the clinico-pathogenesis of this disease, highlight the aggressive nature of this genetic mutation (c.290C>A; p.Ser97Tyr), and document the response to the latest advances in treatment currently available.

## Introduction

Hereditary transthyretin amyloidosis (hATTR) is an autosomal dominant, adult-onset disease that occurs due to point mutations in the TTR gene encoding the protein transthyretin. Classically observed to cause peripheral neuropathy, this condition is additionally associated with extracellular amyloid deposits in multiple organs including the heart, kidney, skin, eyes, and gastrointestinal tract. The disease is progressive and life-threatening with a survival of about five years from the time of disease onset.

Although once considered endemic to a few regions such as Portugal, Sweden, and Japan during the 1990s, the disease is now prevalent in more than 25 countries around the world. The disease has an incidence of approximately 1 in 100,000 persons in the US population, a number that is expected to increase with the broader use of genetic testing and the growing awareness among clinicians. Herein, we present a rare and aggressive variant of hATTR (Ser97Tyr mutation) leading to progressive disease [1-4].

## Case presentation

Patient information and clinical findings

A 45-year-old Caucasian male with no significant past medical history and no current medication use at home presented to the clinic with complaints of significant lower extremity weakness, requiring assistance to walk, and a weight loss of 40 pounds. The patient’s symptoms started around six months prior when he began having numbness and pain in his hands and feet. The patient additionally experienced progressive lower extremity weakness. He had no symptoms of carpal tunnel syndrome or lower back pain. Apart from his neuropathy, the patient had issues with low blood pressure and dizziness, which was notably exacerbated while getting up in the morning. He started noticing swelling in his feet and ankles a few weeks prior to presentation. Physical examination was notable for a prominent fourth heart sound, lower extremity edema, and motor strength of 3/5 in his lower extremities with a diminished fine touch sensation (Table 1). The family history is noteworthy, particularly since his 49-year-old brother, who experienced similar symptoms, recently passed away, with a post-mortem autopsy disclosing the presence of systemic amyloidosis.

**Table 1:** Timeline of patient’s clinical symptoms/findings

Time point	Clinical symptoms/findings
Six months prior to presentation	Numbness and pain in hands and feet, progressive lower extremity weakness, symptoms of low blood pressure, and dizziness
Few weeks prior	Swelling in feet and ankles
Presenting episode	Significant lower extremity weakness, motor strength of 3/5 in lower extremities, diminished fine touch sensation, weight loss of 40 pounds, prominent fourth heart sound

Diagnostic assessment

**Figure 1:**
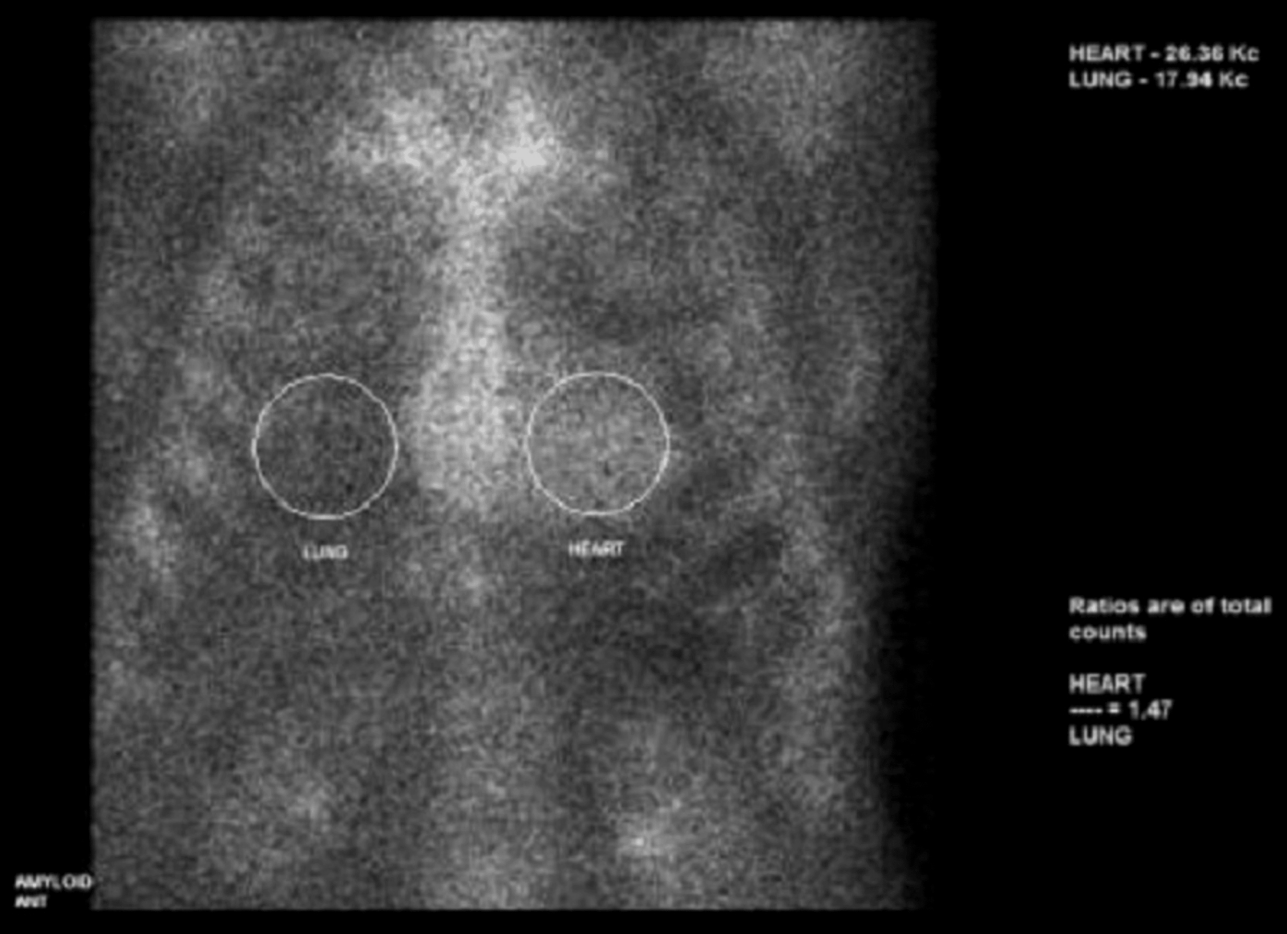
Tc-99 m PYP scan showing increased H/CL uptake ratio Tc-99 m PYP: technetium-99 m pyrophosphate; H/CL: heart-to-contralateral

The patient underwent a neurological workup including imaging, which was inconclusive. Labs were non-significant with no paraprotein detected on immunofixation, and a normal kappa and lambda free light chains (FLC), and kappa/lambda FLC ratio. Echocardiogram was notable for severe concentric left ventricular hypertrophy (LVH), with an increased interventricular and posterior wall thickness, hyperdynamic left ventricular systolic function with an ejection fraction of 70%, normal valvular function, and a moderate circumferential pericardial effusion. Technetium-99 m pyrophosphate (Tc-99 m PYP) scintigraphy showed an equivocal two-and-a-half hour heart-to-contralateral lung (H/CL) ratio of 1.47 and visual grade 1 which was also equivocal for transthyretin amyloidosis (Figure 1).

**Figure 2:**
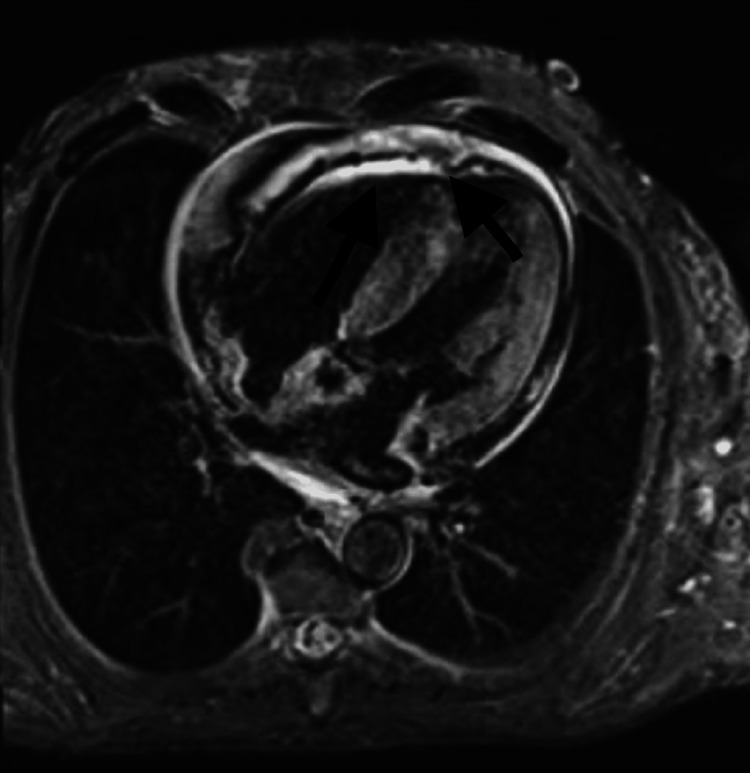
cMRI showing diffuse LGE consistent with amyloid (black arrows) cMRI: cardiac magnetic resonance imaging (cMRI); LGE: late-gadolinium enhancement

**Figure 3:**
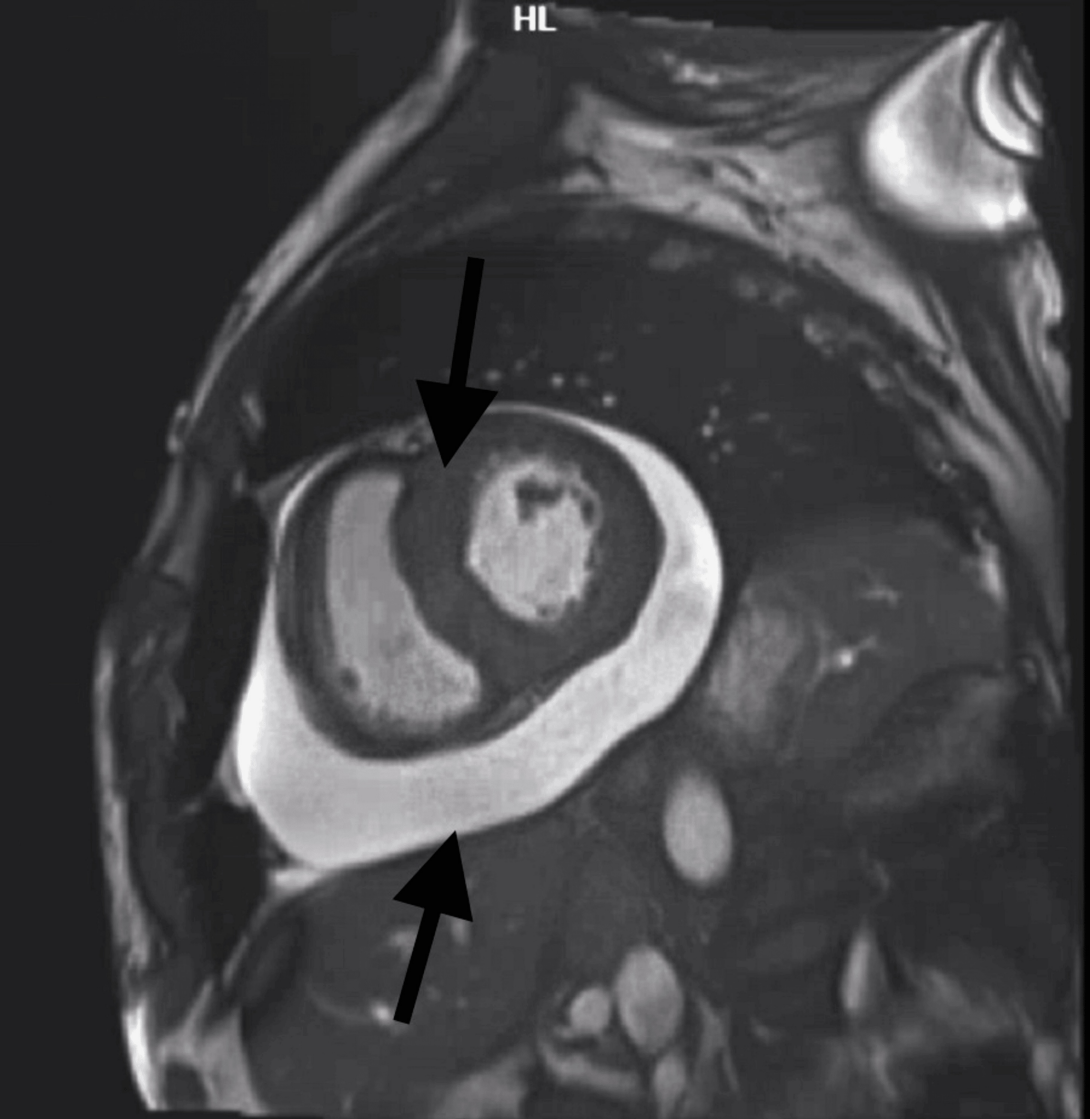
cMRI showing severe LVH and pericardial effusion (black arrows) cMRI: cardiac magnetic resonance imaging; LVH: left ventricular hypertrophy

Cardiac magnetic resonance imaging (cMRI) exhibited a thickened interatrial septum, severe LVH particularly at the base, mid septum, and posterior lateral walls with a maximal wall thickness of 1.7 to 2 cm. cMRI further showed diffuse and patchy left ventricular myocardial delayed hyperenhancement, along with prolonged T1 and T2 mapping scores, subendocardial delayed hyperenhancement throughout the right and left atria, diffusely patchy through the mildly thickened right ventricular myocardium, and increased extracellular volume (Figures 2-3). Further work-up on the patient with genetic testing revealed a variant identified on the TTR gene as the c.290C>A; p.Ser97Tyr mutation, consistent with a diagnosis of hereditary transthyretin amyloidosis.

Therapeutic intervention

Treatment was initiated with patisiran therapy (30mg every 21 days), along with vitamin A supplementation, and tafadamis therapy (61mg daily) to enhance the quality of life.

Follow-up and outcomes

Treatment was well-tolerated with the patient demonstrating a remarkable response with gradual resolution of symptoms and imaging confirming the same. PYP scan one year after treatment initiation demonstrated a H/CL ratio of 1.35 with a visual grade (visual semi-quantitative grading scale analysis) of 0 indicating no myocardial uptake of tracers in the presence of normal rib and bone uptake. Repeat echocardiogram two years following treatment initiation was notable for normal left ventricle (LV) size, and hyperdynamic systolic function, with a left ventricular ejection fraction (LVEF) of 70%. There was moderate concentric hypertrophy with a normal right ventricle (RV) size and systolic function. Most importantly, there was a significantly reduced global longitudinal strain with differential apical sparring. A concurrent PYP scan revealed an improvement in the H/CL ratio to 1.15 (Table 2).

**Table 2:** Outcome assessment through PYP scans PYP: pyrophosphate; H/CL: heart-to-contralateral

PYP scan	Visual grade	H/CL ratio
At the time of diagnosis	1	1.47
1 year following treatment	0	1.35
2 years following treatment	0	1.15

## Discussion

Genotypic and phenotypic heterogeneity is a characteristic hallmark of hereditary transthyretin amyloidosis. More than 130 variants have been identified in the TTR gene consequent to missense mutations. Val30met is the most prevalent mutation that has a predominant neurologic phenotype [3]. The c.290C>A; p.Ser97Tyr variant observed in our patient (also referred to as Ser77Tyr by alternative nomenclature) was documented in a handful of families previously, and had a mixed phenotype [4]. A German family with the same mutation was noted to present with peripheral neuropathy, cardiomyopathy, and renal insufficiency. Cardiomyopathy was also seen in other families from Northern France and Texas [5,6]. Apart from heart failure, other clinical attributes in our patient include peripheral neuropathy, and autonomic neuropathy (orthostatic hypotension).

The average age of onset for ATTR amyloidosis was found to vary between 20 to 90 years. A study of 1865 patients with ATTR amyloidosis in the Portuguese population disclosed a mean age of 52 years. On average, Portuguese populations were noted to have an early onset of disease (<50 years), whereas Japanese populations were noted to have a later onset of disease (>50 years) [7]. Delayed or incorrect diagnosis due to heterogeneity in clinical presentation is common among the patients. Furthermore, it’s a challenge to diagnose in non-endemic regions [1,4].

Multimodality imaging can aid in the diagnosis of amyloidotic cardiomyopathy. Thickening of the right ventricle wall and interatrial septum detected on an echocardiogram are important diagnostic clues characteristic of amyloidosis. In contrast, observation of the granular, speckled appearance of the myocardium and hypertrophic cardiomyopathy lacks specificity. Circumferential pericardial effusion, LV wall thickness >1.5 cm, right ventricle wall thickening, and dysfunction are indicative of a poor prognosis [8-10].

Cardiac MRI when compared to echocardiogram, is a more accurate form of imaging and is especially helpful when echocardiography is limited by poor acoustic windows in obese patients. In cases of low cardiac amyloid burden, as defined by the extracellular volume (ECV) on cMRIs, global longitudinal strain (GLS), and E/e' emerged as the echocardiographic parameters with a high likelihood of being abnormal. Cardiac amyloidosis is characterized by a distinct pattern of GLS with a “cherry on top” bullseye plot. It features significant debilitation of the basal and mid segments with preservation of the apical region. Additionally, the ratio of E/e' may rise above 15, coherent with elevated LV filling pressures. Phelan et al. quantified the relative apical longitudinal strain and revealed a ratio of >1 to exhibit a sensitivity of 93% and specificity of 82% in distinguishing cardiac amyloidosis from other conditions. Post-gadolinium enhancement patterns in cMRIs can be utilized as a distinguishing factor between AL and ATTR amyloidosis. A transmural late-gadolinium enhancement (LGE) was shown to be associated with ATTR amyloidosis. Furthermore, studies have demonstrated transmural LGE to be a predictor of mortality [8-10].

Nuclear imaging can be helpful in diagnosing particularly when echocardiographic and cMRI findings are inconclusive. Tc-99 m-labeled pyrophosphate is one of the most commonly used radiotracers. Its uptake in the myocardium is associated with greater than 99% sensitivity for transthyretin amyloidosis irrespective of the grading. Tc-99 m PYP scintigraphy is highly accurate for the diagnosis of transthyretin cardiac amyloidosis (ATTR CA), with a positive predictive value of 100% when concomitant serum and urine studies for light-chain AL amyloidosis (AL CA) are negative. Heart-to-contralateral (H/CL) uptake ratio can be employed to distinguish from AL amyloidosis wherein a ratio >1.5 is more suggestive of transthyretin amyloidosis [8-10].

When the probability of amyloidosis is still high despite a negative or unequivocal PYP scan, as noted in our patient, tissue samples can be collected for histological confirmation. A biopsy can be falsely negative, sometimes requiring the need for additional biopsies to demonstrate the presence of amyloid. Abdominal fat, nerves, labial salivary gland, and gastrointestinal tract are commonly biopsied. The sensitivity of each type of tissue in detecting the amyloidogenic protein can also show variability. The final step involves the identification of the causative mutation by employing DNA sequencing [11].

Pharmacological strategies targeting the mutated TTR protein encompass TTR stabilizers (tafamidis) and reduction of TTR synthesis (patisiran, vutrisiran, inotersen, and eplontersen). While tafamidis works by stabilizing its tetramer that impedes further deposition in the tissues, patisiran and vutrisiran hinder TTR synthesis by means of disrupting the mRNA with small interfering RNA and inotersen with antisense oligonucleotide [12,13]. Tafamidis was demonstrated to significantly improve quality of life and functional capacity with a reduction of all-cause mortality and cardiovascular-related hospitalizations. However, a subgroup analysis comparing patients with hereditary and wild-type transthyretin amyloidosis revealed a relatively sub-par benefit in terms of cardiovascular-related hospitalizations for individuals with hereditary amyloidosis when compared to those with the wild-type [14,15]. Tafamidis is currently the only FDA-approved medication for ATTR cardiomyopathy. The efficacy of patisiran in the management of both peripheral and autonomic polyneuropathy was demonstrated with a reduction of both hereditary and wild-type amyloid synthesis. The APOLLO Phase 3 study has illustrated significant improvements in LV wall thickness, LV end-diastolic volume, cardiac output, N-terminal prohormone of brain natriuretic peptide (NT-pro BNP), GLS in the basal region, and an improvement in the 10-minute walk test in the patisiran group compared to the placebo [16]. Patisiran, vutrisiran, and inotersen are currently FDA-approved for polyneuropathy of hATTR patients and there are ongoing trials to determine their efficacy in TTR-cardiomyopathy patients [12,13]. This case highlights the remarkable response to treatment with tafamidis and patisiran, with a consequent improvement in the H/CL ratio together with the symptomatology and thus, the prognosis of the patient.

## Conclusions

Our case highlights the aggressive nature of this mutation (c.290C>A; p.Ser97Tyr) as it can lead to symptom manifestation at a relatively younger age and rapid clinical deterioration after symptom onset. Early genetic testing can aid in the prompt initiation of treatment considering the time-sensitive nature of this condition. This case presents a valuable opportunity to elucidate the clinico-pathogenesis of this rare genetic mutation and emphasize the positive response to the latest advances in treatment currently available, thus raising awareness among healthcare professionals.

## References

[1] Manganelli F, Fabrizi GM, Luigetti M, Mandich P, Mazzeo A, Pareyson D (2022). Hereditary transthyretin amyloidosis overview. Neurol Sci.

[2] Adams D, Algalarrondo V, Polydefkis M, Sarswat N, Slama MS, Nativi-Nicolau J (2021). Expert opinion on monitoring symptomatic hereditary transthyretin-mediated amyloidosis and assessment of disease progression. Orphanet J Rare Dis.

[3] Ando Y, Coelho T, Berk JL (2013). Guideline of transthyretin-related hereditary amyloidosis for clinicians. Orphanet J Rare Dis.

[4] Sekijima Y (1993). Hereditary transthyretin amyloidosis. GeneReviews.

[5] Wallace MR, Dwulet FE, Williams EC, Conneally PM, Benson MD (1988). Identification of a new hereditary amyloidosis prealbumin variant, Tyr-77, and detection of the gene by DNA analysis. J Clin Invest.

[6] Planté-Bordeneuve V, Lalu T, Misrahi M, Reilly MM, Adams D, Lacroix C, Said G (1998). Genotypic-phenotypic variations in a series of 65 patients with familial amyloid polyneuropathy. Neurology.

[7] Inês M, Coelho T, Conceição I, Duarte-Ramos F, de Carvalho M, Costa J (2018). Epidemiology of transthyretin familial amyloid polyneuropathy in Portugal: a nationwide study. Neuroepidemiology.

[8] Velaga J, Liew C, Choo Poh AC, Lee PT, Lath N, Low SC, Bharadwaj P (2022). Multimodality imaging in the diagnosis and assessment of cardiac amyloidosis. World J Nucl Med.

[9] Scheel PJ 3rd, Mukherjee M, Hays AG, Vaishnav J (2022). Multimodality imaging in the evaluation and prognostication of cardiac amyloidosis. Front Cardiovasc Med.

[10] Patel RK, Fontana M, Ruberg FL (2021). Cardiac amyloidosis: Multimodal imaging of disease activity and response to treatment. Circ Cardiovasc Imaging.

[11] Kittleson MM, Maurer MS, Ambardekar AV (2020). Cardiac amyloidosis: Evolving diagnosis and management: a scientific statement from the American Heart Association. Circulation.

[12] Stern LK, Patel J (2022). Cardiac Amyloidosis Treatment. Methodist Debakey Cardiovasc J.

[13] Macedo AV, Schwartzmann PV, de Gusmão BM, Melo MD, Coelho-Filho OR (2020). Advances in the treatment of cardiac amyloidosis. Curr Treat Options Oncol.

[14] Maurer MS, Schwartz JH, Gundapaneni B (2018). Tafamidis treatment for patients with transthyretin amyloid cardiomyopathy. N Engl J Med.

[15] Rapezzi C, Elliott P, Damy T (2021). Efficacy of tafamidis in patients with hereditary and wild-type transthyretin amyloid cardiomyopathy: further analyses from ATTR-ACT. JACC Heart Fail.

[16] Adams D, Gonzalez-Duarte A, O'Riordan WD (2018). Patisiran, an RNAi therapeutic, for hereditary transthyretin amyloidosis. N Engl J Med.

